# Preparation and Characterization of Biochars Obtained from Biomasses for Combustible Briquette Applications

**DOI:** 10.1155/2022/2554475

**Published:** 2022-12-06

**Authors:** Chaimaa Hadey, M. Allouch, M. Alami, F. Boukhlifi, I. Loulidi

**Affiliations:** ^1^Engineering Sciences and Trades Laboratory, ENSAM, Moulay Ismail University, Meknes, BP 15290, Morocco; ^2^Laboratory of Chemistry and Biology Applied to the Environment, Faculty of Sciences, Moulay Ismail University, BP 11201-Zitoune, Meknes, Morocco

## Abstract

Renewable energies have been considered as alternative, clean, available, and ecological sources of energy. The production of biochar from biomass by thermochemical means is considered an efficient method of converting biomass for energy production. In this study, the biochars were produced from the biomasses of peanut shells and sugar cane bagasse at different pyrolysis temperatures (400°C, 450°C, and 500°C). The biomass samples and their produced biochars were characterized using calorific value, Fourier transform infrared spectroscopy (FTIR), scanning electron microscopy and energy dispersive X-ray spectrometry (SEM and EDX), compressibility index, and combustion behavior in order to analyze their potential. Experimental results showed that biochar has better fuel qualities compared to raw biomass. We also found that increasing the pyrolysis temperature clearly improved the calorific value, the morphology, the porosity of the biochars as well as the compressibility index of the biochars. The interest of this study was to produce renewable biochar from peanut shell waste and sugar cane bagasse for use as solid fuel.

## 1. Introduction

Energy appears to be one of the most crucial and very important commodities for the sustainability of modern society, international politics, the economic spectrum, and the environment [1].

The large consumption of fossil fuels leads to global warming, the energy crisis, threats to human health, pollution of the oceans, and many other environmental problems [2, 3]. These problems push us to find alternative renewable energy sources, sustainable, clean, inexpensive, and respectful of the environment [1, 4].

Biomass is a form of renewable energy considered respectful of the environment thanks to the use of residues and the elimination of waste [5], it is also considered as one of the carbon neutral resources, available and less expensive [6, 7].

Thermochemical and biological technologies are the simplest technologies currently available for converting biomass to bioenergy [7, 8], in this regard, thermochemical technologies are more efficient and faster and popular for processing biomass compared to biological technologies [9, 10].

Pyrolysis is a process of thermochemical conversion of biomass where it thermally decomposes the structure of biomass into carbon-rich solids (biochar) in an inert environment without oxygen [11, 12].

Biochar is considered to be an environmentally friendly, high calorific solid material, stable fuel, and a good fuel that can be used in many multidimensional applications such as wastewater treatment, reduction of greenhouse gases, energy production, improvement of soil health, agriculture-related activities, and as building materials [13, 14].

Biochar from pyrolysis is an amorphous form of carbon that consists of numerous carbon compounds and ash [15].

The condensed aromatic nature of biochar is what makes it so stable in the environment [16]. Biochar has a very high carbon content, or it can go according to Gaskin et al., from 400 g·kg^−1^ à 900 g·kg^−1^ [17].

The objectives of this study were to produce biochars by pyrolysis based on biomass of peanut shells (PS) and sugar cane bagasse (SC) and to study the effect of different pyrolysis temperatures on the physical properties-chemical, energetic and structural. In order to evaluate their usefulness, it is very important to know the elementary and approximate composition, and then a thermo gravimetric analysis was carried out in order to evaluate the influence of the pyrolysis temperature on the combustion behavior of the samples as well as the indices fluidity (Carr's compressibility index (CCI) and Hausner ratio (HR) the associated fuel quality indices (FR, CI, and VI), and the bulk density of the biochar produced were studied. In addition, the energetic properties such as HHV, LHV, and FVI were calculated. X-Ray diffraction (XRD), Fourier transform infrared spectroscopy (FTIR), and surface morphology (SEM) analysis are useful tools for the characterization of biomass and their biochars. According to the results, it will be possible to compare the two biomasses with their biochars and estimate which would potentially be used as fuels for the manufacture of combustible briquettes.

## 2. Materials and Methods

### 2.1. Raw Materials and Preparation of Biochars

The biomass waste from peanut shells and sugarcane bagasse was used as raw material in the experiments of this work to produce the biochars; the samples were dried and stored before being transformed into biochar. The biochars were prepared by the pyrolysis process in a muffle furnace at a heating temperature of 400°C, 450°C, and 500°C at a heating rate of 20°C.

Biochars prepared at 400°C, 450°C and 500°C, respectively, are presented in PS_400_, PS_450,_ and PS_500_ for biochars derived from peanut shells, and in SC_400_, SC_450,_ and SC_500_ for biochars prepared from sugar cane bagasse. The raw material samples and their biochars were analyzed and characterized in order to observe the effect of pyrolysis temperature on the chemical, energetic and structural characterization.

### 2.2. Biochar Yield

The biochar yield was determined as the ratio of the mass of the biochar product to the mass of the biomass, using the following equation (18):

(1) $$\begin{array}{c} \left(biochar\right)Yield\left(\%\right)=\frac{mass\;of\;biochar}{mass\;of\;biomass}\times 100\text{.} \end{array}$$

### 2.3. Physical and Chemical Characterization

#### 2.3.1. Bulk Density

The bulk density for the biomass samples and their biochar was calculated using the method of Wang and Kinsella using a graduated cylinder, filled with a known quantity of powdered samples which had been dried in the oven and weighed. Then, the cylinder was tapped for almost 1–2 minutes in order to compact the sample, and the sample volume was recorded in ml and bulk density was calculated using equation (2). The tapped density was determined in the same way, except that the cylinder containing the powder was tapped for a fixed number of (50). It was determined by equation (3) [19].

(2) $$\begin{array}{c} bulk\;\;de\;nsity\left({gm}^{-1}\right)=\frac{weight\;of\;\;dr\;y\;material\left(g\right)}{the\;volume\;of\;\;dr\;y\;material\left(ml\right)}\text{,} \end{array}$$

(3) $$\begin{array}{c} Tapped\;\;de\;nsity\left({gm}^{-1}\right)=\frac{weight\;of\;powde\;r\left(g\right)}{the\;tapped\;volume\;of\;powde\;r\left(ml\right)}\text{.} \end{array}$$

#### 2.3.2. Compressibility Index

In order to study the compressibility behavior of the powder mixture according to Lumay et al., the Carr index was determined from the bulk density and the tapped density by using equation (4) (while the Hausner ratio (HR) was calculated using equation (5) [20, 21].

(4) $$\begin{array}{c} {Carr}^{\prime }s\;inde\;x\left(CCI\right)=\frac{Tapped\;\;de\;nsity-bulk\;\;de\;nsity}{Tapped\;\;de\;nsity}\times 100\text{,} \end{array}$$

(5) $$\begin{array}{c} Hausner\;Ratio\left(HR\right)=\frac{tapped\;\;de\;nsity}{bulk\;\;de\;nsity}\text{.} \end{array}$$

#### 2.3.3. Combustion Indices

To assess the utility of biomass samples and their biochar as a solid fuel, we calculated the fuel ratio (FR), combustibility index (CI), and volatile flammability (VI) according to Conag et al. using the following equations [22]:

(6) $$\begin{array}{c} Fuel\;Ratio=\frac{Fc}{V_{m}}\text{,}\\ Combustibility\;inde\;x=\frac{HHV}{\left(FR-115-Ash\right)}-\frac{1}{105}\text{,}\\ Volatile\;ignitability=\frac{\left(HHV-0.338\times Fc\right)}{Vm+Mc}\times 100\text{.} \end{array}$$

#### 2.3.4. Proximate Analysis

Proximate analysis was carried out to obtain the values of ash content; volatile matter (*VM*) and fixed carbon. Volatile matter (*VM*) of feedstock and biochars was determined according to the ASTM standard method D3173 [23], by a sample mass difference before and after combusting 1g of samples placed in an open crucible at 900°C. Concerning the ash content according to the ASTM standard method 7582-10 [24], approximately 1 g for oven-dried samples were heated at 750°C for at least 5 hours, and the percent ash content was determined by measuring the weight difference before and after combustion. Fixed carbon content (FC) was calculated by the following equation:

(7) $$\begin{array}{c} \left(Fixed\;carbon\;content\right)Fc=100\left(Vm+Ash+Mc\right)\text{.} \end{array}$$

#### 2.3.5. Elemental Analysis

The ultimate analysis to determine the C, H, O, and S elemental composition of the feedstock and biochar was determined following the ASTM E777, E778 standard method.

#### 2.3.6. Fourier Transform Infrared Spectroscopy (FTIR)

The functional groups in the biomass as well as biochars were obtained through FTIR measurements using a JASCO 4100 FTIR spectrometer. The powdered samples were well mixed with KBr, and then all IR spectra of the samples were recorded over a wavenumber plage between 400 cm and 4000 cm, or the scanning speed used to detect the FTIR of the samples was maintained at a constant 1 cm. with a resolution factor of 4.

### 2.4. Morphological Analysis

#### 2.4.1. SEM Analyzes/EDS

The surface morphology of biomass and biochars were examined using the Hitachi S-3400N scanning electron microscope at an acceleration voltage of 15 kV. The energy dipersivex-ray spectroscopy (EDS) analysis was performed to determine the elemental composition of the samples.

#### 2.4.2. X-Ray Diffraction Analysis

XRD is used to identify the crystalline phases formed for the biomass samples as well as for the prepared biochars, using the difractometer (Bruker D8 Advance), equipped with the K*α* radiation of copper (*λ* = 1.5406) produced at 50 kV and 20 kV. The 2*θ* scan was scanned between 10 and 65°C with a step size of 0.02 in a compaction time of 20 s.

### 2.5. Thermal Analysis

#### 2.5.1. ATG/DTG

Thermogravimetry (TG) analysis for biomass and biochar samples was carried out using (DTG-60) analyzer. The samples were heated from room temperature to 1000°C under combustion conditions, with a constant heating rate of 20°C/min. The mass loss (TG) curves and differential thermo gravimetric (DTG) curves, were obtained as a function of time and temperature for the reaction conditions examined. In TG-DTG curves, the characteristic temperatures of samples were determined including the starting temperature *Ts*, the ending temperature *Te*, the max temperature *T* max, and the maximum mass loss rate *W* max.

### 2.6. Energy Characterization

#### 2.6.1. HHV

The higher heating value HHV was calculated using the unified correlation HHV from the volatile matter (*Vm*) and fixed carbon (*Fc*) of fuels [25].

(8) $$\begin{array}{c} HHV=0.1846Vm+0.352Fc\text{.} \end{array}$$

#### 2.6.2. LHV

According to Soils, the lower heating value (LHV, kJ/kg) was calculated from HHV, and hydrogen content (H) and oxygen content (O) by the following equation [26]:

(9) $$\begin{array}{c} LHV=HHV-0.212H-0.0080\text{.} \end{array}$$

#### 2.6.3. FVI

From the lower heatingvalue (LHV) determined previously and the apparent density (*ρ*) as well as the ash (*AC*) and humidity contents (*Mc*), according to Mierzwa–Herztek et al., the fuel energy density of the samples was calculated by the following equation [27]:

(10) $$\begin{array}{c} FVI=\frac{LHV*\rho }{Ac*Mc}\text{.} \end{array}$$

#### 2.6.4. EDR and EY

The energy density ratio and energy yield were calculated using the following equations [28, 29]:

(11) $$\begin{array}{c} Energy\;\;de\;nsity\;ratio\left(EDR\right)=\frac{{HHV}_{biochar}}{{HHV}_{biomass}}\text{,} \end{array}$$

(12) $$\begin{array}{c} Energy\;yield\left(EY\right)=\left(biochar\;yield\left(\%\right)\right.\times \frac{{HHV}_{biochar}}{{HHV}_{biomass}}\text{.} \end{array}$$

## 3. Results and Discussion

### 3.1. Biochars Yield

Table 1 shows the biochar yields from peanut shells and sugarcane bagasse at different pyrolysis temperatures. Biochar yield decreased with increasing pyrolysis temperature due to biomass decomposition and thermal degradation of lignocellulosic structures and increased fixed carbon substances [16, 30, 31].

### 3.2. Physical and Chemical Properties

#### 3.2.1. Proximate Analysis of Biochar

Table 2 shows the approximate analysis of biochar obtained from PS and SC biomass at pyrolysis temperatures of 400°C, 450°C, and 500°C. The results show a decrease in the volatile content of PS and SC biochars with increasing pyrolysis temperature shows. This is in agreement with several researchers who have concluded that this decrease may be due to the thermal decomposition of cellulose and lignin and degradation of cellulose, hemicellulose fraction, and noncarbon combustible components of biomass during charring of destruction [32, 33].

The ash content of PS and SC biochars increased with increasing carbonization temperature, which was expected because the increased devolatilization during pyrolysis resulted in a large amount of carbon in the chars. Several authors have reported similar results for different biomasses [34, 35]. Low-temperature briquettes may be more suitable for combustion due to low ash content.

Ash represents the inorganic fraction that cannot be volatilized or degraded by combustion, and generally, the increase in ash is due to the gradual concentration of inorganic components and the reduction of other elements during pyrolysis. Fixed carbon increased from 34.06% to 31.04% for PS_400_ and SC_400_ to 43.32% and 39.58% for PS_500_ and SC_500_^,^ respectively. An increase in the fixed carbon content of the biochar was observed after increasing the pyrolysis temperature, as the devolatilization increases with the increase in temperature, which is preferable because it contributes more to the heat of the energy for the application of biochar as fuel.

#### 3.2.2. Elemental Analysis

Elemental analysis and molar ratios based on elemental analysis of biochars are shown in Table 2. The results show that compared to raw samples biochar has a high carbon content and a low oxygen and hydrogen content. O and H contents decrease in the biochar as the pyrolysis temperature increases. Due to dehydration, decarbonylation, and decarboxylation reactions [31, 36].

The aromaticity index of biochar is indicated by the H/C ratio which followed the same downward trend (pyrolysis temperature increased H/C ratio decreased), a low value of H/C ratio indicates that the biochar produced became more aromatic and more carbonaceous as well as a presence of a graphite-like structure [37, 38].

Molar O/C ratios can be used to indicate biochar polarity. The results show that the O/C ratio decreases with increasing temperature. The lower O/C values of biochar at low temperatures indicated that the surface of biochar at low temperatures may have fewer polar functional groups [34].

Low H/C and O/C ratios produce less CO_2_, smoke, and water vapor when burned, leading to higher combustion efficiency where biochars were acceptable for use as solid fuel.

#### 3.2.3. Density

Using (2) and (3), the bulk and tapped densities of the raw biomasses and their biochars were calculated and presented in Table 3. For the biochar samples, a decrease in bulk and tapped density was also observed with increasing pyrolysis temperature, as well as the bulk and tapped density values for raw biomass were less dense than their biochars. However, raw peanut shells and their biochars were denser than raw bagasse sugar cane and their biochars.

#### 3.2.4. Flowability

From the density values obtained, the flow properties of biomass and pyrolyzed biomass, such as Hausner's ratio (HR) and Carr's compressibility index (CCI), are listed in the table to study the ease of movement of the material and its mobility. Regarding the Hausner ratio (RH), are considered durable tablets since the HR values did not exceed 1.6 [20, 21, 39]. While the CCI values indicate that the samples have good fluidity and are easy to compact [22, 28]. Based on the results found, values of 134.25 for RH and CCI, respectively, were found to be suitable for briquetting and burning applications due to their low fluidity and easy compaction.

#### 3.2.5. Combustion Indices

Combustion indices such as fuel ratio (FR), combustibility index (CI), and volatile index (VI) are determined and presented in Table 4 to provide the quality and performance of biomass and biochars prepared by pyrolysis.

The fuel ratio of raw biomass PS and SC is 0.27 and 0.21, respectively. After pyrolysis, PS400-500 and SC400^−^500 biochars were shown to have an RF ranging from 0.60 to 0.55 to 0.95 and 0.82, respectively, an increase observed with increasing temperature from pyrolysis, linked to the reduction in volatiles and the increase in fixed carbon. These values are within the recommended range for the fuel ratio (0.5–3.0) and make biochars suitable for combustion in power plants [29, 40].

According to Gaskin et al. the combustibility index must be of a value of 23 MJ/kg, According to the results of CI, it can be concluded that the biochars prepared at 500°C can be adapted as an energy source compared to the other biochars and their raw biomass [17].

Biochars were determined to have IVs ranging from 17.60 to 18.05, while raw biomasses of 17.03 and 17.09 for PS and SC, respectively, were these values are in the inflammation range, which must be greater than 14.5 MJ/kg.

In order to assess the quality of the biomass after pyrolysis, the results of the combustion indices found showed that the biochars produced at the pyrolysis temperature of 500°C can be solid fuels and used as an energy source and in other industrial applications.

### 3.3. Energy Properties

The calorific value HHV is an important parameter to assess fuel quality and demonstrate its potential as a feasible option for energy production, the calorific value of produced biochars is affected by pyrolysis temperature and type load [1, 41].

In this study, the calorific values HHV of peanut shell and sugarcane biochars increased with increasing pyrolysis temperature. The increase in calorific values of biochars is attributed to the pyrolysis process which results in the release of volatile matter and an increase in fixed carbon and hence a higher degree of carbonization. High calorific values are required in high-quality fuels in order to exploit as much energy as possible from the briquette fuels.

**Figure 1:**
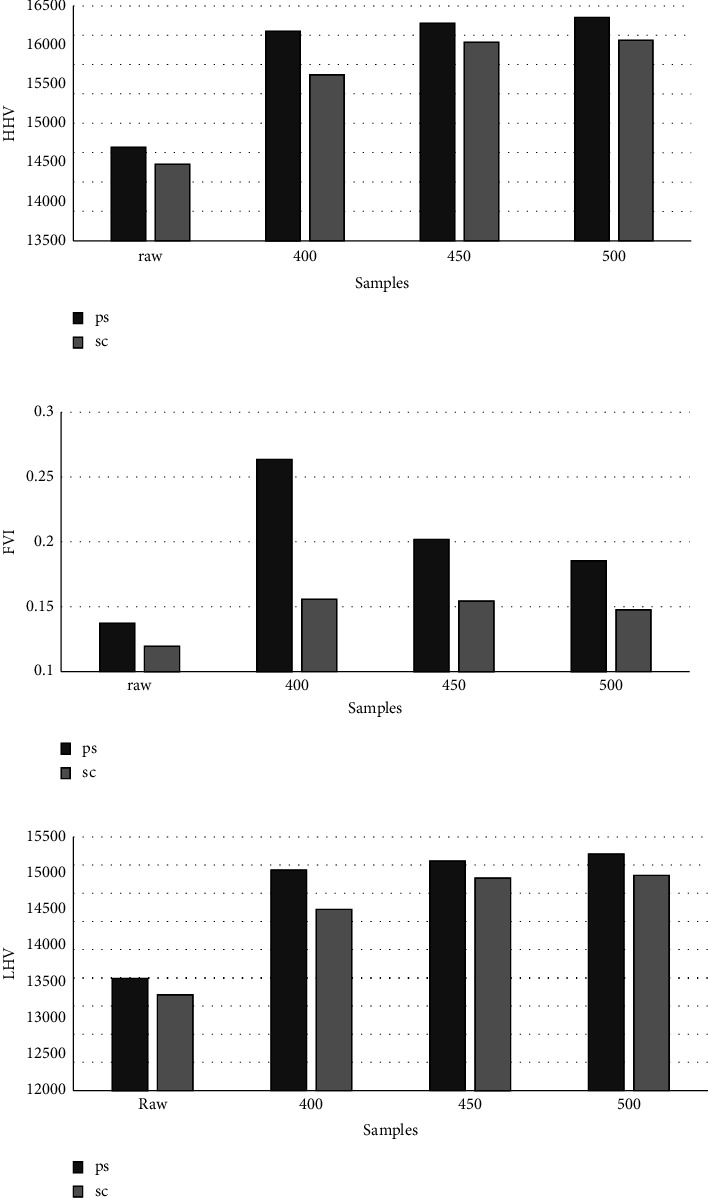
Energy properties of raw and pyrolysis biomass.

From the results presented in Figure 1, by increasing the pyrolysis temperature, an increase in the lower calorific value is observed, this result can be attributed to the low H content, and according to Pariyar et al., the lower H content promotes an increase in PCI [38].

Comparing the two raw samples, the LHV of PS and SC reached the values of 12993.07 and 12704.86, respectively. While the LHV of the biochars of both samples were higher than those of the crude biomasses. The LHV of biochars can consider biochars as biofuels that can be applied in other thermochemical processes.

In Figure 1, the FVI results for the samples are shown. The FVI of PS was higher than that of SC, this result can be attributed to the high calorific value and bulk density of PS. Biochars have higher FVI values than raw biomass. However, by increasing the pyrolysis temperature, a decrease in the FVI is observed, which is due to the reduction in the apparent density.

The energy yield makes it possible to determine the quantity of energy retained in the biomass after pyrolysis. According to the results presented in Figure 1, a decrease in energy yield is observed for both samples with increasing pyrolysis temperature, which is due to the decrease in mass yield [42].

### 3.4. Thermal Degradation Behavior of Biomass and Biochar Samples

**Figure 2:**
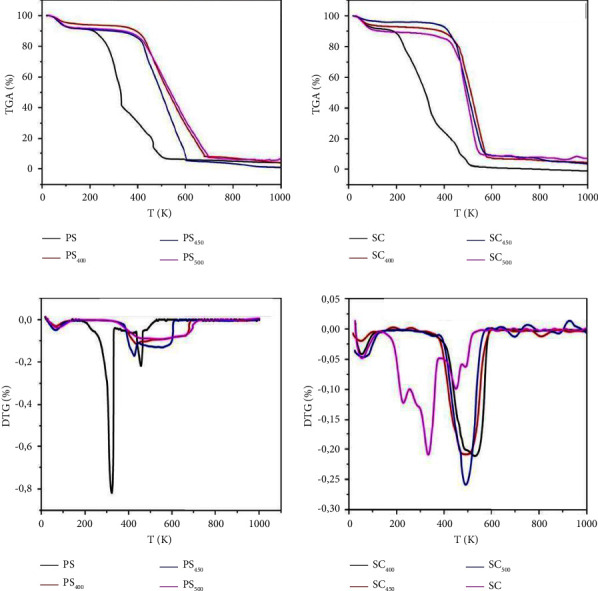
ATG/DTG curves of PS, PS_400_, PS_450_, PS_500_ and SC, SC_400_, SC_450_, SC_500_.

In order to study the combustion behavior and thermal stability of solid biofuels, the thermo gravimetric (TG) and thermo gravimetric (DTG) profiles from biomass and biochar under combustion conditions at a heating rate of 20°C/min are presented in Figure 2.

As shown in the figure, the thermal decomposition process of the biomass samples was classified into three main stages. The first stage of weight loss was apparent between 25°C and 120°C for PS and SC, related to the dehydration process, then a second step between 200°C and 400°C is linked to the devolatilization and decomposition of cellulose and hemicellulose. The third stage was in the range of 400–600 matter decomposition, lignin decomposition, and charcoal combustion.

On the other hand, biochar samples at different pyrolysis temperatures showed only two stages of weight loss during combustion, according to Figure 2. The first stage occurred between 30°C and 130°C attributed to the dehydration process, and for the second stage, the temperature range was 420°C to 530°C, characterized by a large peak at 437.68, 425.84, 544.67, 526.46, 487.73, and 489.79, respectively, for PS_400_, PS_450_, PS_500_, SC_400_, SC_450_, and SC_500_, which is attributed to coal combustion.

### 3.5. Morphological Analysis

#### 3.5.1. SEM Analyzes/EDS

The morphology of the biomass surface and their biochars after pyrolysis was determined by SEM analysis coupled with an energy-dispersiveX-ray (EDX) device. The morphology of biochars has shown a remarkable difference in surface area compared to raw biomass.

**Figure 3:**
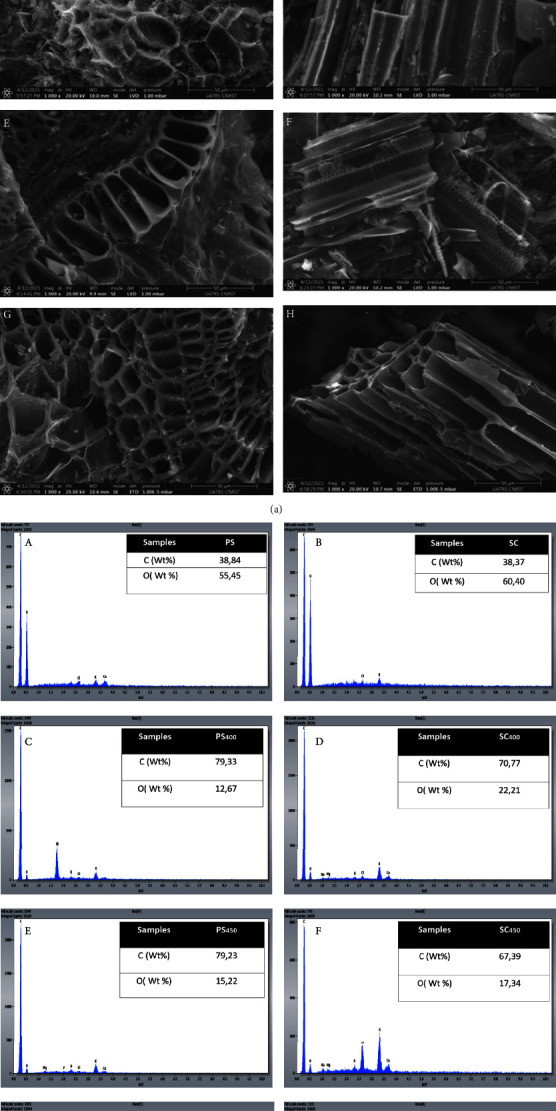
(a) SEM analysis of the peanut shell and bagasse sugar cane derived biochars (A) PS (B) PS_400_, (C) PS_450_, (D) PS_500_, (E) SC, (F) SC_400_, (G) SC_450_, and (H) SC_500_. (b) EDS analysis of the peanut shell and bagasse sugar cane derived biochars (A) PS (B) PS_400_, (C) PS_450_, (D) PS_500_, (E) SC, (F) SC_400_, (G) SC_450_, and (H) SC_50_.

The SEM images presented in Figure 3(a) showed that the biomass had a closed and soft structure and almost negligible pores. Whereas the morphology of the biochars showed that the porosity and the rigidity improved with the increase in the pyrolysis temperature, due to the presence of voids generated by the thermal decomposition of the biomass and the release of volatile matter during pyrolysis [32]. The pyrolysis temperature influences the structure of biochars where the structure has become a more orderly shape because the number of macropores increases while the number of micropores decreases. Biochars (SC) had a smoother and more fibrous surface while biochars from (PS) had larger pores and were well-defined at different pyrolysis temperatures [33, 41].

EDS spectra (Figure 3(b)), showed that all samples are mainly composed of a large amount of carbon (C), oxygen (O), and traces of other elements such as Ca, K, Si, Al, Mg, Cl, P, and Na in very small quantities. These low fractions confirm the low ash contents [42].

PS and SC have a lower amount of carbon than the biochar samples. In addition, the amount of carbon increases with increasing pyrolytic temperature for both samples in the analyzed location.

#### 3.5.2. X-Ray Diffraction Analysis (XRD)

**Figure 4:**
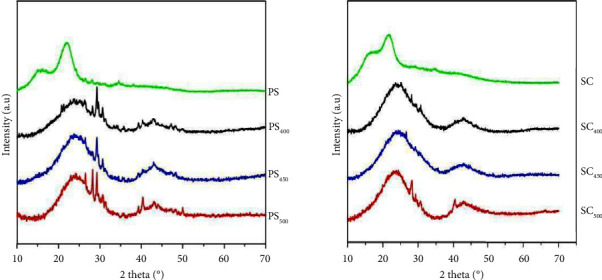
Diffractograms of PS, PS_400_, PS_450_, PS_500_ and SC, SC_400_, SC_450_, SC_500_.

X-ray diffraction is a technique allowing to study of the crystallinity and the amorphism of the biomass and the structure of the biochar obtained by pyrolysis at different temperatures. The XRD curves for the two samples of biomass PS and SC and their biochars are presented in Figure 4.

According to the diffractograms obtained, it was found that the raw biomasses do not have the same structure as their biochars. However, two large peaks were detected at the value 2*θ* between 16 and 25 for the two raw biomasses PS and SC which indicates an amorphous and crystalline cellulose structure [11, 34].

Whereas by increasing the pyrolysis temperature the two peaks disappear and the XRD curves of the biochars present a single peak due to the degradation of the cellulose and the volatilizations of the organic compounds.

This indicates that with the increase in carbonization temperature, the biochars obtained were of carbon-rich amorphous nature, which is consistent with other observations in the literature [35, 38].

#### 3.5.3. The Fourier Transform Infrared (IR)

**Figure 5:**
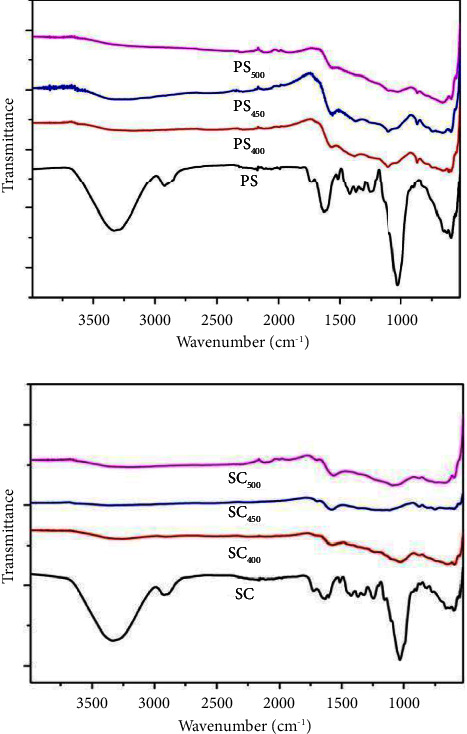
FTIR spectra curves of PS, PS_400_, PS_450_, PS_500_ and SC, SC_400_, SC_450_, SC_500_.

The Fourier transform infrared spectra for the samples studied are represented in Figure 5, by comparing the biomass samples with the biochars obtained at different pyrolysis temperatures, the spectra show that all the biomass and biochar samples have similar functional groups: hydroxyl group, carbonyl group, aromatic and alcohol rings, esters [43]. But it was observed that the pyrolysis temperature had an effect on these functional groups where several peaks disappeared in the spectra of biochars.

The peak obtained in the region between (3000–3700) corresponds to the OH stretch of water molecules of hydroxyl groups (OH) and phenols [11], this peak was observed in all samples of biomass but not in biochars where this disappearance of the OH group could be due to the dehydration of the components of cellulose and hemicellulose and to the release of volatile matter and moisture content during the pyrolysis process [38, 39].

The peak between (2800–2980) correspond to aliphatic C–H stretching vibration. However, the CH_2_ group was identified in the entire spectra as CH_2_ peaks range between 2915 cm^−1^ and 2935 cm^−1^. The small peaks found in this region for the biochar samples could be attributed to the thermal degradation of the cellulose. Cellulose and hemicellulose allowed the destruction of aliphatic structures. The peaks between (1600 and 1800) are associated with a stretching of the C-O rings and the vibration of aromatic C = C valence but these peaks were not observed in the spectra of biochars due to the decomposition of volatile matter during the pyrolysis process. Finally, the peak at 600–800 was noted as the aromatic C-H stretch.

This study has made it possible to obtain biofuels with better combustion characteristics, high calorific values, good fluidity, and combustion indices adapted to combustion, which allow them to be used as solid fuels for heating and cooking at the household level as well as industrial applications in boilers.

## 4. Conclusion

This paper highlighted the effects of the pyrolysis temperature on the energetic, physicochemical and microstructural characteristics of biochars obtained from biomass (peanut shell (PS) and sugarcane bagasse (SC) for briquette applications, the results showed the following conclusions.

The physicochemical analysis methods (IR, RX, elemental and proximate analysis, density) showed that the biochar properties were assessed and found to be improved compared to the properties of the feedstock biomass.

The energetic properties of HHV and LHV of biochars were improved by increasing the pyrolysis temperature, while FVI was reduced with increasing pyrolysis temperature.

TG curves indicated that three steps are responsible for weight loss for crude biomass, while two steps were noted for biochars.

SEM images showed an increase in pore number and stiffness in biochars compared to raw biomass and with increasing pyrolysis temperature.

The results found in this work indicated that both peanut shell biomass and sugarcane bagasse meet the criteria for biochar production, as well as peanut shell biomass (PS) exhibited better properties, compared to sugarcane bagasse (SC). However, the pyrolysis temperature had an important effect on the yield and on the energetic, morphological, and physicochemical characterization of the biochar, therefore its results make the biochar obtained at high temperatures a material that will have satisfactory performance as a solid fuel. [44]

## Figures and Tables

**Table 1:** Yield of peanut shell and bagasse sugar cane biochar samples prepared at different pyrolysis temperatures.

Samples	Yield (%)
PS_400_	36
PS_450_	32
PS_500_	29

SC_400_	25
SC_450_	21
SC_500_	18

**Table 2:** Proximate and ultimate analysis of raw and pyrolysis biomass.

Analysis	PS raw	PS-400	PS-450	PS-500	SC-raw	SC-400	SC-450	SC-500
Proximate analysis (Wt %)								
Moisture content	7.01	5.44	5.35	5.04	7.1	5.66	4.7	4.4
Volatile matter	66.4	56.6	50	45.3	69	56	50	48.02
Ash content	8.59	3.8	5.16	6.34	9.1	7.2	7.3	8
Carbon fixe	18	34.06	39.49	43.32	14.8	31.04	38	39.58

Ultimate analysis (Wt %)								
C	41.67	47.51	47.90	48.20	40.82	45.31	46.95	47.06
H	5.052	5.28	5.15	5.06	5.04	5.09	5.07	5.03
O	37.07	37.32	35.80	35.73	37.34	36.12	35.35	34.88
H/C	0.12	0.11	0.10	0.10	0.12	0.11	0.10	0.10
O/C	0.88	0.78	0.74	0.72	0.91	0.79	0.75	0.74

**Table 3:** Bulk density and flowability indices of raw and pyrolysis biomasses.

Samples	Bulk density (g/ml)	Tapped density (g/ml)	Carr's compressibility index (CCI%)	Hausner ratio
PS raw	0.26	0.32	18.75	1.23
PS_400_	0.34	0.40	15	1.17
PS_450_	0.28	0.35	20	1.25
PS_500_	0.27	0.31	12.90	1.14
SC raw	0.15	0.17	11.76	1.13
SC_400_	0.20	0.28	28.57	1.4
SC_450_	0.19	0.24	20.83	1.26
SC_500_	0.17	0.21	19.04	1.23

**Table 4:** Combustion indices of raw and pyrolysis biomass.

Samples	FR (kg/kg)	CI (MJ/kg)	VI (MJ/kg)
PS	0.27	69.49	17.03
PS_400_	0.60	39.42	17.60
PS_450_	0.78	30.63	17.67
PS_500_	0.95	25.54	17.81
SC	0.21	84.68	17.09
SC_400_	0.55	39.99	18.05
SC_450_	0.76	30.50	17.83
SC_500_	0.82	28.17	17.95

## Data Availability

No data were used to support this study.

## Nomenclature

*Fc*: Fixed carbon:
*Vm*: Volatile matter
*Mc*: Humidity content
*Ac*: Ash content
HHV: Higher heating value
LHV: Lower calorific value
FVI: Fuel energy density
EY: Energy yield
ED: Energy density ratio
FR: Fuel ratio:
CI: Combustibility index
VI: Volatile flammability
*CCI*: Carr's index
*HR*: Hausner ratio
*ρ*: Apparent density.
